# A multiscale characterization of cortical shape asymmetries in early psychosis

**DOI:** 10.1093/braincomms/fcae015

**Published:** 2024-01-22

**Authors:** Yu-Chi Chen, Jeggan Tiego, Ashlea Segal, Sidhant Chopra, Alexander Holmes, Chao Suo, James C Pang, Alex Fornito, Kevin M Aquino

**Affiliations:** School of Psychological Sciences, Turner Institute for Brain and Mental Health, and Monash Biomedical Imaging, Monash University, Melbourne 3800, Australia; Monash Biomedical Imaging, Monash University, Melbourne 3800, Australia; Monash Data Futures Institute, Monash University, Melbourne 3800, Australia; Brain and Mind Centre, University of Sydney, Sydney 2050, Australia; Brain Dynamic Centre, Westmead Institute for Medical Research, University of Sydney, Sydney 2145, Australia; School of Psychological Sciences, Turner Institute for Brain and Mental Health, and Monash Biomedical Imaging, Monash University, Melbourne 3800, Australia; Monash Biomedical Imaging, Monash University, Melbourne 3800, Australia; School of Psychological Sciences, Turner Institute for Brain and Mental Health, and Monash Biomedical Imaging, Monash University, Melbourne 3800, Australia; Monash Biomedical Imaging, Monash University, Melbourne 3800, Australia; Department of Psychology, Yale University, New Haven, CT 06511, USA; Department of Psychology, Yale University, New Haven, CT 06511, USA; School of Psychological Sciences, Turner Institute for Brain and Mental Health, and Monash Biomedical Imaging, Monash University, Melbourne 3800, Australia; Monash Biomedical Imaging, Monash University, Melbourne 3800, Australia; School of Psychological Sciences, Turner Institute for Brain and Mental Health, and Monash Biomedical Imaging, Monash University, Melbourne 3800, Australia; Monash Biomedical Imaging, Monash University, Melbourne 3800, Australia; BrainPark, School of Psychological Sciences, Monash University, Melbourne 3800, Australia; School of Psychological Sciences, Turner Institute for Brain and Mental Health, and Monash Biomedical Imaging, Monash University, Melbourne 3800, Australia; Monash Biomedical Imaging, Monash University, Melbourne 3800, Australia; School of Psychological Sciences, Turner Institute for Brain and Mental Health, and Monash Biomedical Imaging, Monash University, Melbourne 3800, Australia; Monash Biomedical Imaging, Monash University, Melbourne 3800, Australia; School of Psychological Sciences, Turner Institute for Brain and Mental Health, and Monash Biomedical Imaging, Monash University, Melbourne 3800, Australia; Monash Biomedical Imaging, Monash University, Melbourne 3800, Australia; School of Physics, University of Sydney, Sydney 2050, Australia; Center of Excellence for Integrative Brain Function, University of Sydney, Sydney 2050, Australia; BrainKey Inc, San Francisco, CA 94103, USA

**Keywords:** early psychosis, spectral shape analysis, cortical asymmetry, eigenmode decomposition

## Abstract

Psychosis has often been linked to abnormal cortical asymmetry, but prior results have been inconsistent. Here, we applied a novel spectral shape analysis to characterize cortical shape asymmetries in patients with early psychosis across different spatial scales. We used the Human Connectome Project for Early Psychosis dataset (aged 16–35), comprising 56 healthy controls (37 males, 19 females) and 112 patients with early psychosis (68 males, 44 females). We quantified shape variations of each hemisphere over different spatial frequencies and applied a general linear model to compare differences between healthy controls and patients with early psychosis. We further used canonical correlation analysis to examine associations between shape asymmetries and clinical symptoms. Cortical shape asymmetries, spanning wavelengths from about 22 to 75 mm, were significantly different between healthy controls and patients with early psychosis (Cohen’s *d* = 0.28–0.51), with patients showing greater asymmetry in cortical shape than controls. A single canonical mode linked the asymmetry measures to symptoms (canonical correlation analysis *r* = 0.45), such that higher cortical asymmetry was correlated with more severe excitement symptoms and less severe emotional distress. Significant group differences in the asymmetries of traditional morphological measures of cortical thickness, surface area, and gyrification, at either global or regional levels, were not identified. Cortical shape asymmetries are more sensitive than other morphological asymmetries in capturing abnormalities in patients with early psychosis. These abnormalities are expressed at coarse spatial scales and are correlated with specific symptom domains.

## Graphical Abstract

**Graphical Abstract:**
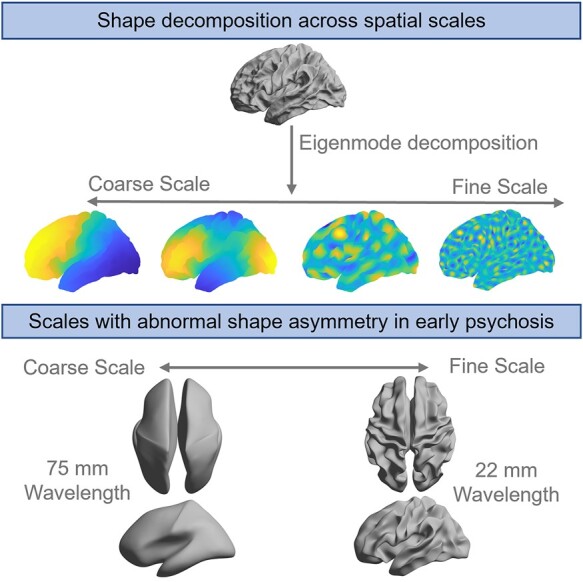

## Teaser

Patients with early psychosis show increased cortical shape asymmetry at coarse spatial scales, while asymmetries of cortical thickness, surface area, and gyrification do not differ between groups. Patients with greater cortical shape asymmetry exhibit more severe impulsivity symptoms and less severe emotional distress.

## Introduction

Relative to other primates, the human cerebral cortex shows greater anatomical and functional asymmetry of the left and right hemispheres, and greater inter-individual variability in this asymmetry.^1-3^ Accordingly, hemispheric asymmetries have been implicated in the evolution of human-specific cognition and behaviour,^4,5^ and abnormal hemispheric lateralization has been linked to psychosis,^6-8^ which may potentially be related to the unique expression of this syndrome in humans.^9^ However, the reported links between abnormal hemispheric asymmetry and psychosis have not been consistently replicated.^7,10-14^

Brain asymmetries are commonly considered on a population basis by mapping average asymmetry levels across a group of individuals,^4,11,15-17^ which is also referred to as directional asymmetry.^2,18^ For instance, the left planum temporale, encompassing Wernicke’s area, is substantially larger, on average, than its right-hemisphere counterpart in healthy controls (HCs).^5^ Interestingly, patients with schizophrenia and their relatives show reduced asymmetry in planum temporale compared to HCs.^8,19,20^ However, population-based studies can obscure the considerable variability in cortical asymmetry that occurs between individuals,^2,3,18,21,22^ with many people showing little or even reversed asymmetries relative to the population average.^2,3,18,23^ Individual deviations of asymmetry from the population mean are referred to as fluctuating asymmetries and may be driven by environmental factors, developmental instability/plasticity, or individual-specific genetic perturbations.^18,22,24-27^ Studies of fluctuating asymmetries in psychosis are scarce, but Núñez *et al*.^28^ have shown that such asymmetries in cortical shape at the global level are increased in patients with schizophrenia and are associated with negative symptoms.

One factor that complicates the identification of reliable asymmetry phenotypes in psychosis is that most analyses to date have focused either on defined regions-of-interest or have relied on point-wise (e.g. voxel-based) analyses, which only consider asymmetries at certain spatial resolution scales.^19,20,29,30^ However, cortical asymmetries can be identified at multiple scales, ranging from entire hemispheres (e.g. Yakolevian torque^31^) to more fine-scale sulcal and gyral features.^32^ Whether abnormal asymmetries in psychosis are expressed at certain specific scales or are a multiscale phenomenon remains unclear. Moreover, most anatomical asymmetry studies use size-related measures, such as volume, cortical thickness, and surface area, which often conflate individual differences in size and shape and have limited sensitivity for capturing individually unique properties of brain anatomy.^21,33^

The ability to isolate the shape effect is critical.^21^ A recent study by Pang *et al*.^34^ showed that the geometrical structure or shape of the cortex constrains human brain functions and connectivity, with this relationship expressed across multiple spatial scales. Mathematically, a spectral description of cortical shape can be obtained through the eigen-decomposition of the Laplace–Beltrami operator (LBO) of the cortical surface.^21,35,36^ The resulting eigenfunctions correspond to an orthogonal basis set of spatial patterns of different spatial frequencies that capture the geometry of the cortex, and the eigenvalues represent their spatial frequencies (see Methods).^33,35,36^ Analysis of these spatial eigenfunctions is ubiquitous in many branches of physics, engineering, and biology^33,35,36^ and naturally captures geometric properties from the coarse scale (low-order eigenfunctions) to the fine scale (high-order eigenfunctions), forming a multiscale description of cortical geometry.^21,35^ This multiscale description departs from conventional methods focusing on specific regions or global hemispheric differences (i.e. they are limited to a single spatial scale).^10,15,19,20,30,37,38^

Building on this concept, our recent study^39^ demonstrated that any empirical map of anatomical variations can be described in terms of these LBO eigenfunctions. Moreover, we have recently shown that multiscale spectral descriptions of asymmetries in cortical shape, rather than size, are highly personalized, akin to a cortical fingerprint, and can identify healthy subjects more accurately than common morphological measures (e.g. volume, cortical thickness, and surface area), measures of inter-regional functional coupling, and the cortical shapes of individual hemispheres.^21^ Moreover, we found that optimal subject identifiability of cortical shape asymmetry occurs at coarse spatial scales, corresponding to wavelengths larger than about 37 mm. We also found that these coarse-scale asymmetries are associated with individual differences in general cognitive function and are largely driven by unique environmental, rather than genetic factors.^21^ Together, these findings suggest that spectral shape analysis of cortical asymmetries offers a window for understanding highly personalized features of brain anatomy.

Here, we applied spectral shape analysis to investigate cortical asymmetries in patients with early psychosis (EP) who were within five years of the initial psychosis onset. EP is a crucial period to understand brain changes associated with the development of psychosis that is less confounded by prolonged treatment exposure.^40^ Prior work has shown abnormal cortical asymmetry in patients with psychosis^19,20,28,29,37^ and that, in healthy individuals, cortical shape asymmetry at coarse spatial scales captures the most individualized and robust information.^21^ Thus, we aimed to examine scale-specific cortical shape asymmetry in EP patients compared to HCs and used the multiscale shape description offered by our method to isolate scale-specific and shape-specific effects that traditional methods based on cortical thickness, surface area, and local gyrification index (LGI) cannot identify. Finally, we explored the relationships between cortical shape asymmetry and different psychotic symptoms.

## Materials and methods

### Neuroimaging data

#### Human Connectome Project for Early Psychosis

We used open-source data from the Human Connectome Project for Early Psychosis (HCP-EP; https://www.humanconnectome.org/study/human-connectome-project-for-early-psychosis^40^), which includes 169 participants with preprocessed structural MRI data. We excluded data for one individual who showed matched asymmetry signature (MAS) values (see details below) that were more than three standard deviations below the sample mean. The remaining 168 participants included 112 patients with EP (aged 16–34, mean = 22.83, standard deviation = 3.75; male = 68, female = 44) and 56 HCs (aged 16–35, mean = 24.90, standard deviation = 4.08; male = 37, female = 19). For EP patients, the chlorpromazine equivalence (CPZ) of the current antipsychotic drug was between 0 and 1000 mg/d (mean = 165 mg/d; standard deviation = 231.52 mg/d), and the exposure time to antipsychotic medication was between 0 and 56 months (mean = 14.3). The patients were diagnosed with non-affective (*n* = 79) and affective (*n* = 33) psychosis and were all within five years of the initial onset of their psychotic symptoms. The criteria for non-affective psychotic disorders included the diagnosis of schizophrenia, schizophreniform, schizoaffective, delusional disorder, psychotic disorder not otherwise specified, and brief psychotic disorder according to the Diagnostic and Statistical Manual of Mental Disorders, Fifth Edition (DSM-V). The criteria for affective psychotic disorders is the DSM-V diagnosis of major depressive disorder with psychosis or bipolar disorder with psychosis.^40^ See Supplementary Table 1 and https://www.humanconnectome.org/storage/app/media/documentation/HCP-EP1.1/HCP-EP_Release_1.1_Manual.pdf for more details.

#### Ethics statement

All participants provided their written informed consent to participate in the HCP-EP, and the HCP-EP was reviewed and approved by the HCP. The HCP-EP complied with the ethical standards of the relevant national and institutional committees on human experimentation and with the code of ethics of the World Medical Association (the Helsinki Declaration of 1975, as revised in 2013).

#### Image acquisition and processing

Imaging data were collected on three Siemens MAGNETOM Prisma 3T scanners, with 32-channel head coils at Indiana University and Brigham and Women’s Hospital, and a 64-channel head and neck coil at McLean, but the neck channels were turned off (https://www.humanconnectome.org/storage/app/media/documentation/HCP-EP1.1/HCP-EP_Release_1.1_Manual.pdf). Special procedures and analyses had been taken to ensure the homogeneity of the image quality across both sites.^40^ The Connectome Coordinating Facility at Washington University provided image processing, central quality control, and data coordination services for all the HCP-style datasets.^41^ The HCP-EP datasets underwent the same protocol as the HCP lifespan dataset.^41^ In brief, the T1-weighted structural MRI scans applied a multi-echo magnetization prepared rapid gradient echo sequence with a high isotropic resolution of 0.8 mm. Other parameters include an echo time of 1000 ms, a repetition time of 2400 ms, and 208 slices. For more details, see Harms *et al*.^41^ and the Human Connectome Project's imaging protocols at https://www.humanconnectome.org/study/hcp-lifespan-aging/project-protocol/imaging-protocols-hcp-aging. The HCP-EP dataset included cortical surface meshes created by applying the FreeSurfer-HCP pipeline,^42-45^ which is based on FreeSurfer version 6.0^43^ with HCP-specific enhancements,^42^ on the T1-weighted MRI images. The cortical surface meshes were further downsampled and registered on the fsLR-32k template, with 32 492 vertices on each hemisphere of the cortex.^46^ The fsLR-32k template provides an accurate cortical shape of the standard Montreal Neurological Institute (MNI) template^42^ but is less computationally demanding than the native MNI surface mesh model.^42^ We used the registered images provided by the HCP-EP dataset without further corrections or smoothing.

#### Clinical and medication assessment

In the HCP-EP dataset, the severity of psychotic symptoms of EP patients is measured by the Positive and Negative Syndrome Scale (PANSS^47^). We followed van der Gaag *et al*.^48^ to employ a five-factor model that has been widely used for evaluating psychotic symptoms.^49-54^ The five-factor model is more robust and clinically relevant than the original three-factor model.^48,52,54^ The five-factor model comprises the following dimensions: positive symptoms, negative symptoms, disorganization symptoms, excitement, and emotional distress, and was constructed by ten-fold cross-validation with more than 5000 subjects.^48^ We used factor analysis with maximum likelihood estimation and oblique rotation^55,56^ as implemented in previous studies^57,58^ to extract the five factors from the items that had occurred across all folds of the cross-validation in van der Gaag *et al*.^48^ To confirm the robustness of our results, we also tested the five-factor model using the items selected by a recent meta-analysis,^59^ yielding similar results.

The HCP-EP dataset provided lifetime exposure duration of antipsychotic drugs and CPZ of current antipsychotic drugs. Both measures were uncorrelated with the MAS eigen-groups in EP patients (*P*_FDR_ > 0.8 and uncorrected *P*-values all > 0.5), suggesting that medication has a limited influence on cortical shape asymmetries.

### Spectral shape analysis

**Figure 1:**
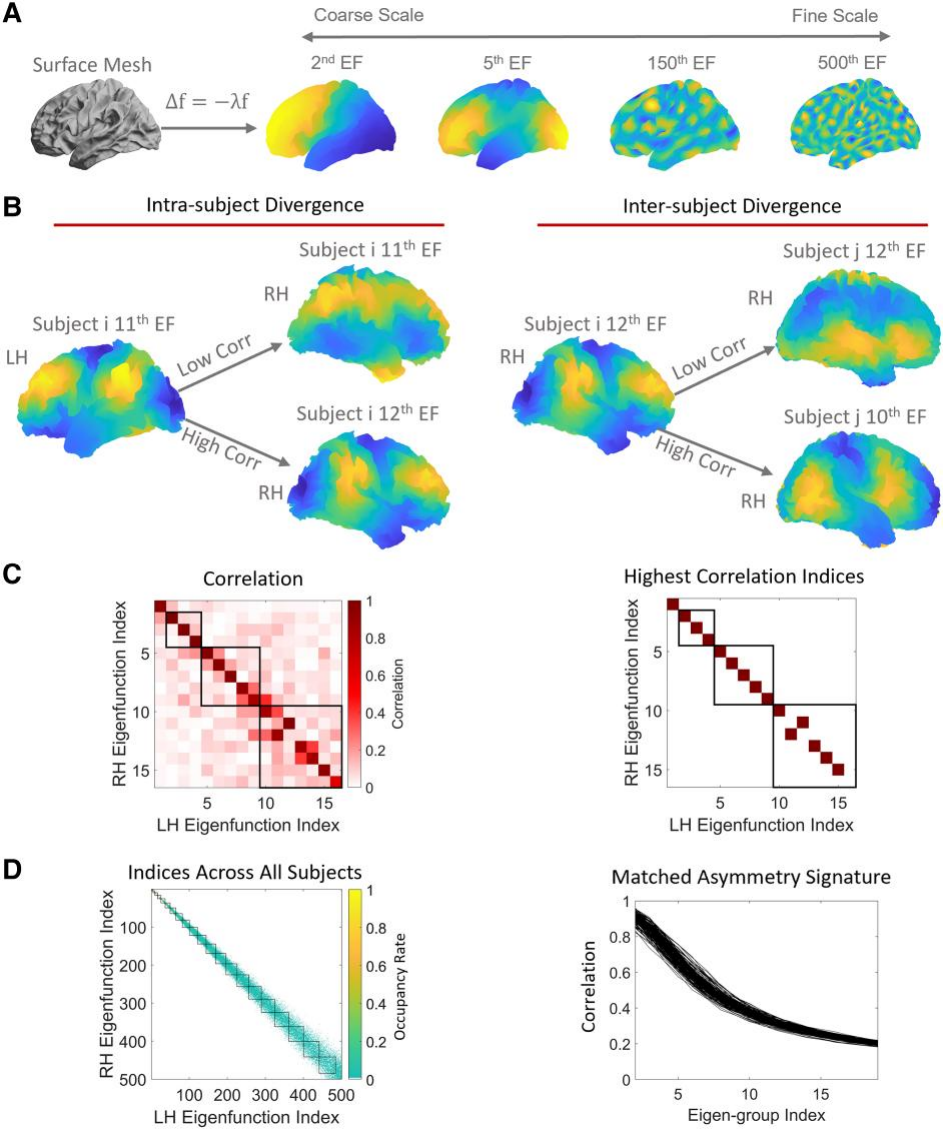
**Schematic of the analysis workflow.** (**A**) The shapes of the left and right hemispheres (surface mesh) were independently decomposed using the eigenfunctions of the LBO. These eigenfunctions describe shape variations at different spatial scales, ordered from coarse (e.g. the 2nd eigenfunction) to fine scales (e.g. the 500th eigenfunction). (**B**) An example of intra- and inter-subject divergences in eigenfunction patterns. The left panel shows that the 11th left eigenfunction is clearly more similar to the same individual’s 12th right eigenfunction than the 11th right eigenfunction. The right panel shows that one individual’s 12th right eigenfunction is more like another individual’s 10th right eigenfunction than the 12th right eigenfunction. (**C**) Left panel, correlations between the left and right eigenfunctions from the individual depicted in the left panel of **B**. From this correlation matrix, we plotted the indices with the highest correlations across all 500 analysed eigenfunctions in the right panel. The coloured squares represent the indices with the highest correlations. Here, we only show the first 16 eigenfunctions, and the squares denote the boundaries between eigen-groups (refer to the section Cortical shape eigen-groups and wavelengths in Materials and methods for details). For each row of the matrix, we selected the maximum absolute correlation. The vector of these correlations across rows is the MAS. (**D**) The left panel presents the occupancy rate, which is the average of the highest indices across all subjects. Most maximal correlations occurred within the same eigen-groups, but some of them appeared in adjacent eigen-groups. Right panel, we calculated the mean of MAS across each eigen-group and its two adjacent eigen-groups. This eigen-group-based MAS condenses the 500 eigenfunction-specific values into 20 eigen-group-specific values. Here, each line represents one individual. The data are from the HCP for EP (*n* = 168). Panel **A** was adapted from Ref. 21.

To obtain a multiscale shape description of the left and right cortical surface meshes obtained from FreeSurfer, we solved the eigenvalue problem of the LBO, which is given by^33,35,36,60^:


(1) Δf=−λf

where Δ is the LBO and *f* is a distinct eigenfunction (EF) with corresponding eigenvalue *λ*. The LBO captures local vertex-to-vertex spatial relations and changes in curvature (i.e. geometry). The eigenfunctions are orthogonal and describe shape variations at different spatial wavelengths, ordered from coarse-grained (the second eigenfunction in Fig. 1A) to fine-grained scales (e.g. the 500^th^ eigenfunction in Fig. 1A).^35,36,61-63^ The eigenvalues form an ordered sequence that ranges from zero to infinity, i.e. $0\leq \lambda^{1}\leq \lambda^{2}\leq \cdots <\infty$, and each eigenvalue is analogous to the wave frequency of sinusoids in Fourier analysis.^21,35,36^ The family of eigenfunctions thus represents an orthogonal basis set that can be used to fully decompose and reconstruct the shape of each cortical hemisphere.^36^ Intuitively, eigenmodes of the LBO can be thought of as preferred ways in which a system can respond to a perturbation. For instance, the functions of a drum head will correspond to its vibration patterns when struck by a stick and the eigenvalues are analogous to the vibration frequency of the drum membrane.^62^

We utilized the Shape-DNA software package^33,35,36^ to perform an eigen-decomposition of the cortical surface of each hemisphere of an individual.^42-45^ Shape-DNA uses the cubic finite element method to solve and optimize the eigenvalue problem of the LBO based on the intrinsic shape of an object or cortex.^35,36^ Shape-DNA outperforms other shape analysis methods for retrieving object shapes^64^ and has been applied to characterize brain shapes with superior subject identifiability than other conventional measures^21,33^ such as cortical volume, thickness, gyrification, and inter-regional functional coupling.^65^

### Matched asymmetry signature

In our previous work, we directly calculated the differences between the LBO eigenvalue spectra of the left and right hemispheres to characterize the fluctuating asymmetry of cortical shape across a spectrum of spatial scales, termed the shape asymmetry signature (SAS).^21^ The SAS is sensitive to quantifying individualized shape asymmetry features^21^ but is too individualized to facilitate group comparisons, as the spatial patterns defined by higher-frequency eigenfunctions are often highly divergent to allow simple pooling across individuals. We therefore developed a new spectral approach for quantifying cortical shape asymmetry that is more suitable for group comparisons, which more accurately accounts for hemispheric differences in the spatial patterning of shape variations. Instead of using the eigenvalues, we calculated the product-moment correlation between the left and right eigenfunctions to characterize the divergence of the two hemispheres within each individual.

A critical challenge in this regard is that the eigenfunctions of the left and right hemispheres are not guaranteed to be directly comparable. As shown in Fig. 1B, eigenfunctions of the left and right hemispheres do not necessarily overlap at the same index (i.e. the maximum correlation may occur at different indices); in this particular individual, the 11th eigenfunction of the left hemisphere is more similar to the 12th eigenfunction of the right hemisphere (*r* = 0.96) than the 11th eigenfunction of the right hemisphere (*r* = 0.17; Fig. 1B, left panel). This occurs because subtle variations in the shape of the left and right cortices can alter the ordering of eigenfunctions, sometimes resulting in quite distinct eigenfunctions at higher spatial frequencies. Although the difference in the order of the left and right eigenfunctions reflects important individual shape variations, this divergence makes it challenging to compare eigenfunctions across individuals.

In this study, after obtaining 500 eigenfunctions for each hemisphere of each individual (Fig. 1A), we first estimated correlations between the spatial pattern of each pair of left–right eigenfunctions, resulting in a symmetric 500×500 correlation matrix quantifying the similarity between the left and right eigenfunctions across spatial scales (e.g. Fig. 1C, left panel). The maximum absolute values of correlations observed across the rows of this matrix (or equivalently, columns) quantify the similarity between optimally matched eigenfunctions of the left and right hemispheres at each of the 500 spatial scales considered in the decomposition (e.g. Fig. 1C, right panel). The resulting vector of 500 maximum correlations thus provides a multiscale, individualized description of cortical shape asymmetries, which we term the matched asymmetry signature (MAS), with lower MAS values corresponding to higher shape asymmetry. We take the absolute value of the correlation because the sign of a given eigenfunction is arbitrary.

### Cortical shape eigen-groups and wavelengths

**Figure 2:**
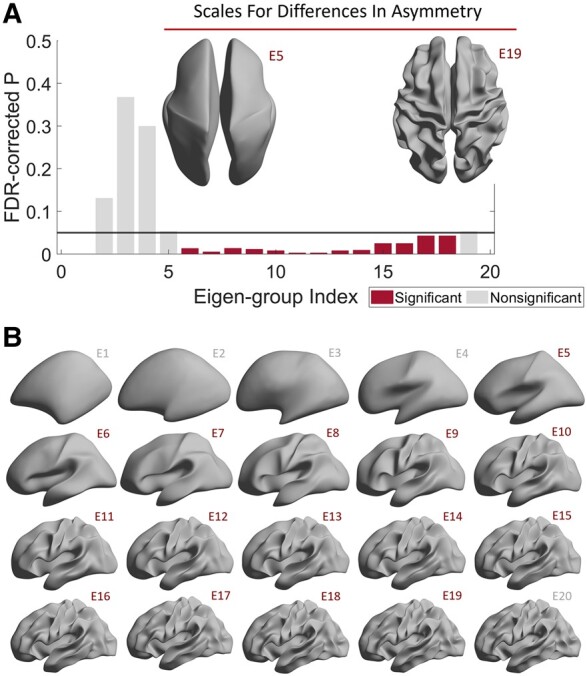
**EP is associated with scale-specific differences in cortical shape asymmetry.** (**A**) The 6th to 18th eigen-groups of the MAS are different between the HC and EP groups. We used a sliding window approach for MAS. Consequently, the wavelengths of the 6th to 18th eigen-groups of the MAS are equivalent to the 5th to 19th original eigen-groups, which are between ∼75 mm (left inset) and ∼22 mm (right inset). The data are from the HCP for EP (*n* = 168), and the cortical surfaces were reconstructed from an open-source and population-based template (fsaverage). (**B**) Cortical surfaces were reconstructed at different spatial wavelengths, starting from the first eigen-group (E1) and incrementally adding more eigen-groups up to the first 20 eigen-groups (E20). Note that these groups are based on the original eigen-groups (not the ones obtained after using the sliding window approach). Panel **B** was adapted from Ref. 21.


In the case of a perfect sphere, the eigenvalues and eigenfunctions of the LBO can be sorted into distinct groups, called spherical harmonics. Within the same spherical harmonic group (i.e. eigen-group), the eigenvalues are degenerate (i.e. identical), and the corresponding eigenfunctions describe shape variations that have similar spatial wavelengths, but which vary spatially along orthogonal axes.^63^ There are 2(*L* + 1) − 1 eigenfunctions in the *L*th group^21,63^: the first eigen-group (*L* = 1) comprises the second to fourth eigenfunctions, the second eigen-group (*L* = 2) comprises the fifth to ninth eigenfunctions, and so on. The first eigen-group describes shape variations at the coarsest spatial scales, which are the variations along the *X*, *Y*, and *Z* axes. Higher eigen-group measures shape variations at finer spatial scales (see Fig. 2B for visualization of the spatial scales of the eigen-groups). It has been shown that these spherical harmonic groups are roughly conserved for the cortex, since the cortex is comparably equivalent to a sphere and has a similar coarse-scale geometry.^63^ We can therefore use an approximation of the spatial wavelength in the spherical case to estimate the corresponding wavelength of each cortical eigen-group^21^:

(2) $$W=\frac{2\pi Rs}{\sqrt{L(L+1)}}$$

where *Rs* is the radius of the corresponding sphere of the original object (*Rs* is about 67 mm for the population-based template, i.e. fsaverage in FreeSurfer) and *L* is the index of the eigen-group. Accordingly, because the ordering of specific eigenfunctions can vary not only within individuals but also between individuals (e.g. Fig. 1B, right panel), it is more appropriate to focus on the groupings of the eigenfunctions, rather than specific eigenfunctions themselves, when considering scale-specific asymmetries.

Figure 1C and D indicates that although most of the highest left-right correlations occur for eigenfunctions from the same eigen-group, some of the maximal correlations are found in adjacent groups. For instance, the right panel of Fig. 1C shows that the 11th eigenfunction of the left hemisphere is maximally correlated with the 12th eigenfunction of the right hemisphere (from the subject in Fig. 1B, left panel). Therefore, the indices of maximal correlations are not entirely on the diagonal line of Fig. 1C. This problem can be addressed using the eigen-group framework because both the 11th and 12th eigenfunctions belong to the 3rd eigen-group (the 3rd black square in Fig. 1C, which encompasses the 10th to 16th eigenfunctions). We thus took the average of the indices with the maximal correlations across all subjects, representing occupancy rates in the left panel of Fig. 1D. For example, if the occupancy rate of the 401st left eigenfunction and the 400th right eigenfunction is 0.2, it means that, for 20% of subjects, the 401st left eigenfunction is most strongly correlated with the 400th right eigenfunction. The left panel of Fig. 1D also shows that the maximal left-right correlations generally occur around the diagonal line and within the same eigen-group (each black square represents each eigen-group) or its two adjacent eigen-groups.

We therefore used a sliding window approach and took the mean of MAS values within each eigen-group and its two adjacent eigen-groups to collapse the MAS from 500 eigenfunction-specific values to 20 eigen-group-specific values. For example, we took the mean correlations across the 1st to 3rd eigen-groups (2nd to 16th eigenfunctions) to represent the 2nd eigen-group of the MAS and the mean across the 2nd to 4th eigen-groups (5th to 25th eigenfunctions) to represent the third eigen-group (see Supplementary Fig. 1 for more details and examples of MAS). In this study, we ignored the first eigen-group because it represents the coarsest spatial scale (∼170 mm wavelength) and is highly conserved, providing little individual-specific information. We also did not consider eigen-groups beyond the 19th, since the mean MAS across all subjects was low (i.e. r<0.2, SD = 0.008), indicating that very fine-scale variations in the shapes of the left and right hemispheres are largely independent. We excluded data for one individual who showed eigen-group-specific MAS values that were more than three standard deviations below the sample mean (see Supplementary Fig. 2 for details).

Previous studies have shown that the shape measures of the LBO are robust to image noise.^21,61^ To confirm the robustness of the MAS to image quality, we used the Euler numbers from FreeSurfer^21,66-68^ to quantify the image quality. We took the mean of the Euler number from the left and right hemispheres and then calculated the Pearson’s correlation between the mean Euler number and the eigen-group-specific MAS values. All eigen-groups of the MAS were unrelated to the Euler number (absolute *r* values < 0.08; *P*_FDR_ > 0.8), confirming that image quality generally does not influence the MAS.

To summarize, we characterize the shape of each cortical hemisphere using the eigenfunctions of LBO, which offers a natural mathematical description of how the shape of an object varies through space. The eigenfunctions correspond to fundamental spatial patterns (akin to axes or modes) of shape variation through space that are intrinsic to each cortical hemisphere and each individual. The corresponding eigenvalues correspond to the spatial frequency, or wavelength of each eigenfunction, and are ordered such that low values correspond to coarse-scale shape variations (e.g. broad anterior–posterior gradients; see Fig. 1A, 2nd EF), whereas high values correspond to fine-scale shape variations (e.g. local sulcal and gyral architecture; see Fig. 1A, 500th EF). The MAS is estimated as the correlation of the spatial pattern between a given eigenfunction in the left hemisphere and the most similar eigenfunction in the right hemisphere. It thus represents the degree of left-right similarity in fundamental patterns of shape variation. Since the cortex is topologically and geometrically comparable to a sphere and sets of eigenfunctions in the spherical case correspond to shape variations with the same wavelength, we can group eigenfunctions into eigen-groups of spatial patterns with similar wavelengths (see Fig. 2B). Averaging across eigen-groups thus provides a more robust index of scale-specific shape asymmetries. Moreover, our approach isolates asymmetries in cortical shapes that are distinct from those in size. This is critical, since shape asymmetries are highly unique to individual brains and are under the influence of unique environmental influences, rather than genetic or common environmental factors.^21^ They thus offer a window into brain changes related to environmental risk factors.

### Size-based anatomical asymmetry

To compare the effects of shape asymmetry to other commonly used morphological asymmetries, including cortical thickness, surface area, and LGI (the ratio between pial surface and outer smoothed surface automatically measured by FreeSurfer^69^), we applied a widely used asymmetry index^16,70,71^ to quantify these non-shape-based morphological asymmetries as:


(3) $$AI^{i}=\frac{M_{L}^{i}-M_{R}^{i}}{0.5(M_{L}^{i}+M_{R}^{i})}$$

where $AI^{i}$ is the asymmetry index of subject *i*, $M_{L}^{i}$ is the mean value (across the whole hemisphere or region) of the morphological measurement, i.e. cortical thickness, surface area, or LGI, from the subject *i*’s left hemisphere, and $M_{R}^{i}$ is the value from the right hemisphere. We registered each subject’s image on the fsLR-32k template^46^ and calculated the asymmetry at both global (i.e. whole hemisphere) and regional levels. For the regional level, we calculated the cortical thickness, surface area, and LGI of each region defined in the Desikan–Killiany^72^ and HCPMMP1^73^ atlases with 34 and 180 regions in each hemisphere, respectively. We excluded the hippocampus from the HCPMMP1 atlas as cortical thickness cannot be measured for this structure.

### Statistical analysis

After calculating the eigenfunctions of LBO from the Shape-DNA algorithm,^35,36^ the resulting eigenfunctions were further analysed using MATLAB (version R2020b). We used a general linear model (GLM) to analyse the differences of each eigen-group between HCs and EP patients as well as between EP patients with non-affective and affective psychosis, controlling for sex, age, neuroimaging sites, and total brain size as confounding variables. We did not control for medication effects because both exposure duration of antipsychotic drugs and CPZ of current antipsychotic drugs were unrelated to all the eigen-groups of MAS considered in this study (*P*_FDR_ > 0.8) in EP patients. We also did not control for handedness because previous studies have found that cortical shape asymmetry is unrelated to handedness.^21,33^

Eigen-groups analysed in this study included their adjacent groups and were not independent of each other. We therefore used a permutation test with 50 000 iterations for statistical inference. We used a tail approximation in comparing the original GLM coefficients with permuted coefficients to ensure reliable *P*-value estimations^74^ and controlled the false discovery rate (FDR, *q* = 0.05) to correct for multiple comparisons.

We examined correlations between the eigen-groups of MAS and PANSS factors using canonical correlation analysis (CCA) on the 104 EP patients who responded to all items of the PANSS.^47^ We first used principal component analysis (PCA) to reduce dimensionality and minimize collinearity of the eigen-groups of MAS at different scales. We then applied the CCA to identify maximal covariance^75^ between the PCs of MAS eigen-groups and PANSS factors by linear combinations, controlling for age, sex, neuroimaging sites, and total brain size as confounding variables. The *P*-values of the canonical modes were calculated using a recently developed CCA permutation procedure^75^ with 50 000 iterations and controlling for family-wise error rate (FWER).^75^ We used bootstrapping with 1000 iterations to identify reliable estimates of loadings of each PANSS factor on the canonical variate,^21,76^ and the resulting *P*-values were then corrected for multiple comparisons by FDR (*q* = 0.05). We also applied bootstrapping with 1000 iterations and FDR correction to measure reliable correlations between each eigen-group of the MAS and the canonical variate.

## Results

### Increased cortical shape asymmetries at coarse scales in EP patients

We first used separate GLMs to compare the 2nd to 19th eigen-groups of MAS between patients and controls. We found that the 6th to 18th eigen-groups (Fig. 2A), spanning wavelengths from about 22 to 75 mm (Fig. 2A insets), were significantly different between the HC and EP groups (*P*_FDR_ < 0.05; Cohen’s *d* = 0.28–0.51; Supplementary Fig. 3), with the MAS in the EP group being significantly lower than the HC group. In other words, patients showed greater asymmetry in shape than controls. The distributions of the MAS of these two groups are shown in Supplementary Fig. 3. Although the 6th to 18th eigen-groups were all significantly different between the HC and EP groups, the effect sizes of some eigen-groups, such as the 11th and 12th eigen-groups (*d* = 0.51 and 0.49, respectively), were higher than other eigen-groups, such as the 17th and 18th eigen-groups (*d* = 0.29 and 0.28, respectively; Supplementary Fig. 3). In contrast, we found that commonly used morphological asymmetries, i.e. based on cortical thickness, surface area, and LGI, at both global (GLM *P*-values > 0.05) and regional levels and using either the HCPMMP1 or Desikan–Killiany atlases, were not different between the HC and EP groups (GLM *P*_FDR_ > 0.05). Thus, asymmetries of cortical shape are more sensitive than traditional morphological measures in discriminating HC and EP individuals.

Next, we compared patients with affective and non-affective psychosis. None of the eigen-groups (2nd to 19th) of the MAS showed significant differences between the two groups (*P*_FDR_ > 0.05).

### Cortical shape asymmetries correlate with symptom severity

We used CCA^75^ to examine relationships between the eigen-groups of MAS and the five factors of psychotic symptoms measured by the PANSS. We applied PCA to the 6th to 18th eigen-groups, which showed differences in our group analysis (see Fig. 2), and retained the first two PCs, which explained 92% of the variance.

**Figure 3:**
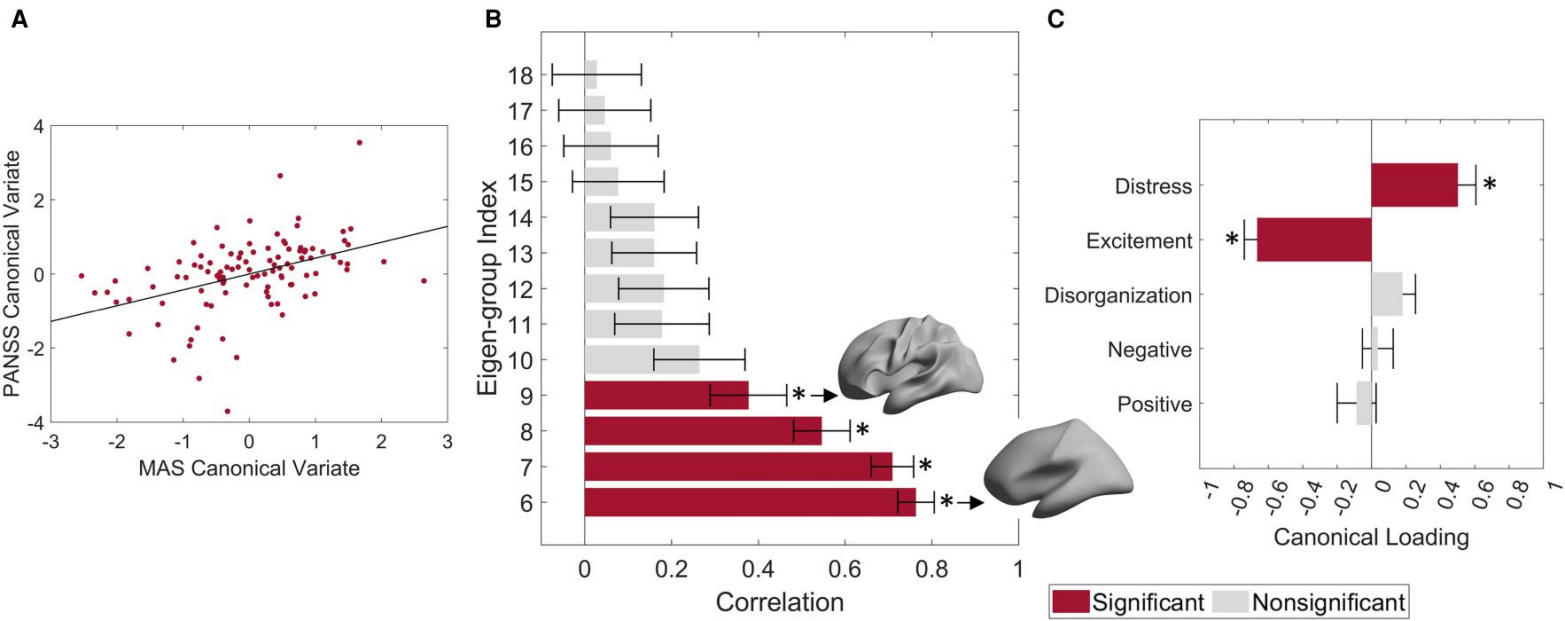
**Individual differences in the MAS are correlated with symptom severity.** (**A**) We performed a CCA between the canonical variates of the first two principal components of the MAS eigen-groups and the five PANSS factors. The first canonical mode of this CCA yielded a significant correlation of 0.45 (CCA *r* = 0.45; *P-*value controlled for family-wise error rate = 0.002). The solid line represents the least-squares regression line. (**B**) Pearson’s correlations between the 6th to 18th eigen-groups of the MAS and the corresponding canonical variate. We found the 6th to 9th eigen-groups to have significant correlations. The insets provide a visual representation of the range of the corresponding spatial wavelengths. (**C**) Canonical variate loadings, measured by Pearson’s correlation, of the PANSS factors. We found the loadings on the excitement and emotional distress factors to be significant. For panels **B** and **C**, the error bars represent ±2 bootstrapped standard errors, and the asterisks denote bootstrapped *P*-values corrected by the FDR (*P*_FDR_) < 0.05. The data are from 104 patients with EP in the HCP for EP dataset.

The CCA identified a single canonical mode that was statistically significant (CCA *r* = 0.45; *P*_FWER_ = 0.002; Fig. 3A). The loadings of the first two PCs (0.54 and 0.83) of the MAS on the canonical variate were all significant (*P*_FDR_ < 0.0001), and the 6th to 9th eigen-groups were positively correlated with the canonical variate (*P*_FDR_ < 0.05; Fig. 3B), which spans wavelengths from about 40 to 75 mm (Fig. 3B insets). The loading of PANSS factors was negative (*P*_FDR_ < 0.001) for excitement (representing impulsivity^52^) and positive (*P*_FDR_ < 0.001) for emotional distress (Fig. 3C), indicating that patients with higher cortical shape asymmetry show more impulsivity symptoms and less severe emotional distress. We also confirmed that the antipsychotic drug exposure time was not correlated to all PANSS factors (*P*_FDR_ > 0.2).

## Discussion

Altered brain asymmetries have frequently been reported in patients with psychosis. Here, we applied a multiscale approach to examine abnormalities of cortical shape asymmetry, independent of variations in regional size, in EP patients. We found that EP patients showed a higher degree of cortical shape asymmetry across a range of coarse spatial scales when compared to HC. In contrast, no group differences in asymmetries in cortical thickness, surface area, and LGI were identified. EP patients with a higher cortical shape asymmetry showed more severe excitement and less severe emotional distress. Together, these findings support the sensitivity of our spectral approach for characterizing shape asymmetries in psychosis. They further indicate that altered asymmetries of shape are expressed at specific spatial wavelengths and that individual differences in these asymmetries are related to symptom severity.

### EP patients show increased asymmetry of cortical shape at coarse scales

Surprisingly, we found that EP patients had greater left-right asymmetry of cortical shape when compared to controls. This finding contradicts to many previous studies reporting reduced asymmetries in patients.^19,20,29,30,37^ Conventional asymmetry studies use size-related measures, such as volume or cortical thickness, which can conflate variations in shape and size.^21^ The distinction between shape- and size-based features is crucial because two objects can have similar volumes but with very different shapes.^35,77^ Although the literature on shape asymmetry in patients with psychosis is very limited, one prior study has also found an increased asymmetry of global cortical shape in patients with schizophrenia.^28^ Núñez *et al*.^28^ applied a dice coefficient to measure cortical asymmetry by calculating the ratio of the intersection area between the original cortex (e.g. right cortex) and flipped cortex (new right cortex that was flipped from the left) to the total area of the original cortex and the flipped cortex. However, this approach cannot disentangle the effects of shape from size and cannot measure scale-specific effects. For an extreme example, even if two hemispheres are symmetric in shape, but one hemisphere is proportionally larger than the other, the dice coefficient may be the same as in the case where the two hemispheres are asymmetric in shape but identical in volume. A previous study has shown that isolating shape asymmetry from size effects provides superior subject identifiability.^21^ Unlike the dice coefficient, the MAS disentangles shape asymmetry from the effect of size and decomposes the asymmetry at different spatial scales. Our spectral approach suggests that this increased asymmetry is apparent within a specific spatial wavelength range between ∼22 mm and ∼75 mm. Within this range, the strongest effect size occurred at a spatial wavelength of about 37 mm, while the effect sizes decreased at finer spatial wavelengths. The results are in line with our previous study,^21^ which found optimal subject identifiability of cortical shape asymmetry at a spatial wavelength of about 37 mm.

Our scale-specific approach is akin to analysing the seismic wave frequencies of earthquakes at the global tectonic level, whereas classical point-wise approaches are analogous to only focusing on a specific city,^21^ which may miss broader patterns. This is in line with Pang *et al*.’s^34^ study, which found that human brain function is constrained by the shape of the whole brain at coarse scales. Furthermore, grey matter abnormalities in patients with psychosis are often widespread,^13,78^ although such deviations may show minimal overlap in specific brain locations across patients,^79-81^ potentially reflecting the known clinical heterogeneity of the condition^79,81,82^ or the inaccurate delineation of regional borders.^83^ The vast majority of past work on cerebral asymmetries in psychosis has focused on size-related measures, estimated at global, regional, or point-wise levels,^13,20,29,37^ which are not sensitive to diffuse and heterogeneous brain abnormalities in patients with psychosis. Our analysis found no differences in the asymmetry of size-related measures at global or regional levels, which aligns with other works showing limited evidence for altered asymmetry of these measures in people with psychosis.^11-14,30^ Indeed, the findings have been highly inconsistent, with one meta-analysis showing that even for the superior temporal gyrus—the region most widely reported as showing abnormal asymmetry in patients with schizophrenia—about half of the studies do not find evidence of significantly altered size-based asymmetry.^10^ Similarly, although some studies have reported decreased LGI values in patients with psychosis or those risk groups,^84-86^ the results were not consistently reproduced, with some studies showing increased gyrification or absence of the effects.^30,87-89^

In this study, we found no differences in the LGI asymmetry at both global and regional levels. Our results are in line with previous research showing that intrinsic cortical shape modes derived from the LBO are superior to the LGI in identifying individual variations.^33^ Although the LGI is related to cortical shape, it measures gyrification at the vertex level and the result of no group effects on LGI asymmetry is in line with our MAS finding that the asymmetry effects are mostly found at coarse rather than fine spatial scales. The MAS captures geometric variations across the entire cortex across multiple spatial scales simultaneously and is more sensitive to psychosis-related brain changes than other traditional morphological measures, such as cortical thickness, surface area, or LGI. Our findings thus suggest that the spectral approach developed here offers a new, more sensitive way of understanding asymmetries of cortical shape. Replication of these findings in independent samples will be an important extension of this work.

Our spectral approach offers a natural way of characterizing fluctuating asymmetries, which capture individual-specific brain phenotypes.^21^ Fluctuating asymmetries are hypothesized to arise from developmental instability or individual-specific genetic perturbations^27,28^ and are mainly driven by individual-specific environmental influences.^21^ Many studies have suggested that environmental factors in early life, such as maternal stress, infections, nutrition during pregnancy, childhood adversity, and stress, may affect neurodevelopment and contribute to psychosis.^7,90^ Studies of fluctuating asymmetry in the human brain are limited, but some have found that fluctuating asymmetries of the left-right sides of the human body, such as asymmetry of left and right fingerprints, are also increased in patients with psychosis.^26,91-93^

### Cortical shape asymmetry is correlated with psychotic symptoms

EP patients with higher cortical shape asymmetries at coarse scales showed more severe excitement symptoms and less severe emotional distress. These coarse spatial scales correspond to wavelengths of about 40–75 mm. This range is within the spatial scales previously found to be optimal (larger than 37 mm) for distinguishing individual brains and for identifying correlations with general cognitive function.^21^ Together, these findings suggest that cortical shape asymmetries at coarse scales, but not fine scales, contain personalized brain features which have implications for both normal and abnormal brain functions, including changes in hierarchical functional processing.^94^ Past studies using point-wise approaches have found associations between cortical asymmetries of size-based measures and hallucinations,^38,95^ but the findings have not been consistently replicated. For example, Ohi *et al*.^30^ did not find any correlation between clinical symptoms and asymmetries of volume, thickness, and surface area in the superior temporal gyrus.

The excitement factor of the PANSS may be more related to attention-deficit/hyperactivity disorder (ADHD) and oppositional defiant disorder, while the emotional distress factor is related to anxiety, depression, and stress. Studies have found abnormalities in cortical asymmetry in the above disorders^7,96,97^ and have suggested that various forms of stress are key risk factors for many mental health disorders.^7^ However, we did not find differences in MAS between patients with affective and non-affective psychosis. This result may be due to the small sample size of patients with affective psychosis (*n* = 33).

### Limitations and future directions

In this study, we analysed EP patients to minimize the effects of prolonged medication. Nonetheless, about 78% of the patients had prior exposure to antipsychotics. Although we found that antipsychotic drug exposure time and current CPZ values were unrelated to MAS, the long-term effects of medication on shape asymmetries remain unclear. Future work should aim to replicate our results on medication-naive patients with first-episode psychosis or to compare the results in different psychosis stages, such as chronic schizophrenia. Moreover, it would also be beneficial for future studies to examine a wide range of neuropsychiatric and neurological diseases, such as ADHD, autism, bipolar disorders, and dementia, given that abnormal brain asymmetries have been reported in these diseases.^7,17,98,99^ The MAS also has the potential to measure the development of cortical asymmetry in children and adolescents or its relationships with brain age.

## Conclusion

We developed a novel method to derive a multiscale characterization of cortical shape asymmetries and showed that patients with EP displayed increased asymmetries at coarse scales. In contrast, asymmetries of cortical thickness, surface area, and gyrification were not different between patient and control groups. We also found that patients with higher degrees of cortical shape asymmetries at coarse scales showed more severe excitement symptoms and less severe emotional distress. Together, these findings indicate that cortical shape asymmetries are more sensitive than other morphological asymmetries in capturing differences between patients with psychosis and HCs, and these asymmetry features were related to clinical symptoms.

## Supplementary material

Supplementary material is available at *Brain Communications* online.

## Supplementary Material

fcae015_Supplementary_Data

## Data Availability

All data analysed or generated in this study are included in the manuscript. All code and dependent toolboxes used in this study can be found at: https://github.com/cyctbdbw/MAS_psychosis. The code of Shape-DNA can be found at: http://reuter.mit.edu/software/shapedna/. The HCP-EP dataset is available at: https://www.humanconnectome.org/study/human-connectome-project-for-early-psychosis.
