# Supplementary material for: A multiscale characterization of cortical shape asymmetries in early psychosis
Source: Brain Commun. 2024 Jan 22;6(1):fcae015. doi: 10.1093/braincomms/fcae015 (PMC10859637; doi:10.1093/braincomms/fcae015)
Supplement: fcae015_Supplementary_Data [file fcae015_supplementary_data.pdf]

A multiscale characterisation of cortical shape asymmetries in early psychosis

Supplementary Material

Supplementary Table 1. Demographic and clinical data of the HCP-EP dataset

	Count or Mean ± SD				Group comparison *P*-value	
	HC	EP	Affective	Non-affective	HC Vs. EP	Non-affective vs. Affective
Age	24.90 ± 4.08	22.83 ± 3.75	23.76 ± 4.28	22.44 ± 3.46	0.0013	0.09
Gender (Female %)	33.93 %	39.29 %	63.64%	29.11%	0.50	< 0.001
Site						
IU	24	56	7	49	0.38	< 0.001
BWH	19	26	8	18	0.14	0.87
McLean	13	30	18	12	0.62	< 0.001
Handedness (right-hander %)	80.36%	87.50%	90.91%	86.08%	0.22	0.48
Current CPZ [a]	0	165 ± 231.52	40.63 ± 124.07	216.03 ± 246.20	N/A	<0.001
Lifetime exposure (months) [a]	0	14.30 ± 15.72	11.00 ± 16.52	15.65 ± 15.28	N/A	0.16
PANSS total score [b]	N/A	49.63 ± 10.79	42.69 ± 9.11	52.32 ± 10.22	N/A	< 0.001

[a] Current CPZ (Chlorpromazine Equivalence) and antipsychotic lifetime exposure period of EP patients were from 78 patients with non-affective psychosis and 32 patients with affective psychosis.
[b] Positive and Negative Syndrome Scale (PANSS) scores were from 104 early psychosis (EP) patients, including 75 patients with non-affective psychosis and 29 patients with affective psychosis.
[c] Five PANSS factors were constructed from the factor analysis with items selected by van der Gaag et al..[1]

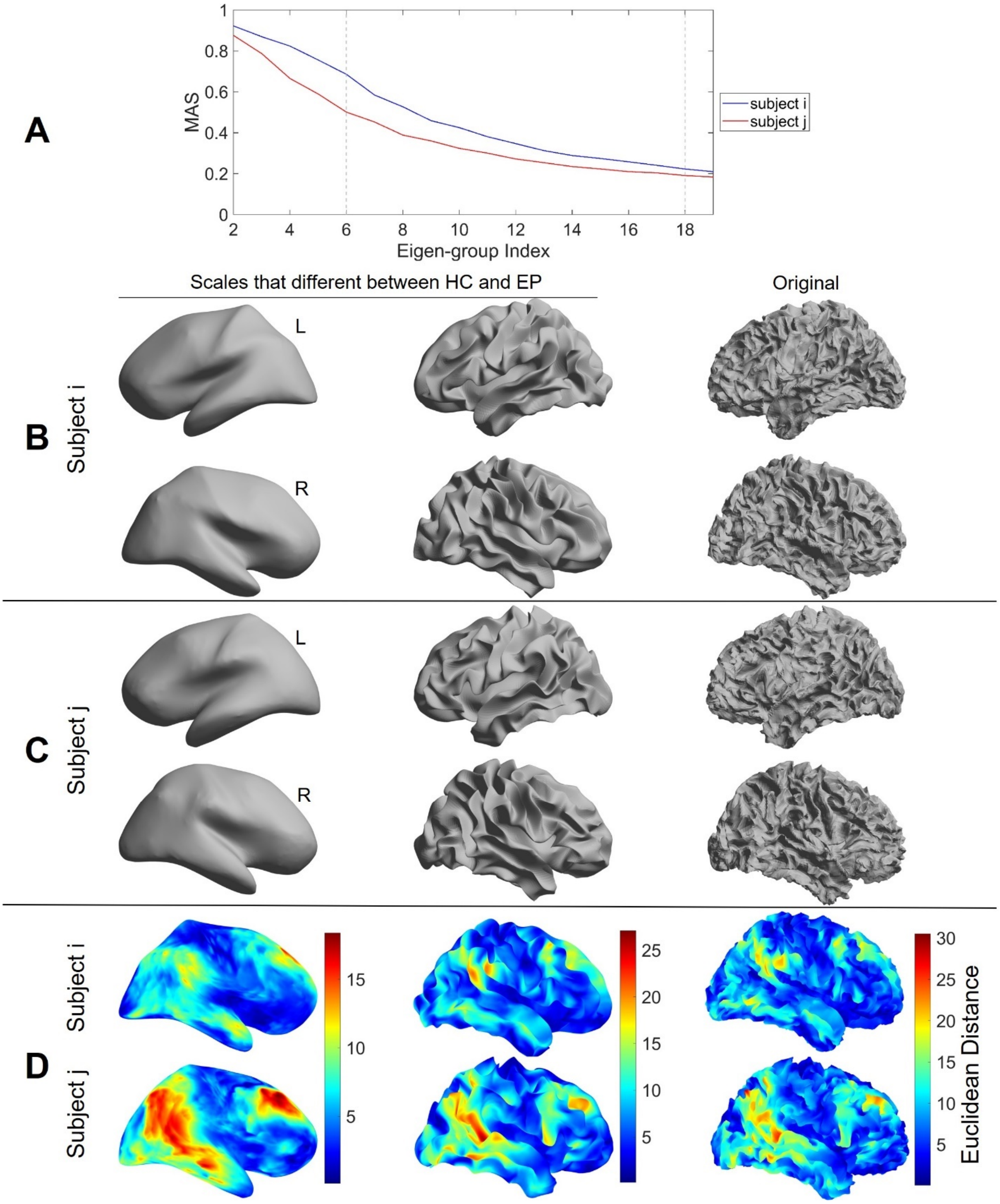

Supplementary Figure 1. Examples of the matched asymmetry signature (MAS) of a healthy control (subject *i*) and a patient with early psychosis (subject *j*). (A) The eigen-groups of MAS of subject *i* (blue line) is higher than those of subject *j* (red line) across spatial scales. The MAS was based on the correlations between the left-right eigenfunctions; therefore, lower MAS values represent lower degrees of left-right similarity and higher degrees of hemispheric asymmetry. **(B)** and **(C)** are the cortical surfaces of subjects *i* and *j* reconstructed at different spatial scales, respectively. The images were all registered on the fsLR-32k

template.[2] The spatial scales that showed significant differences between HC and EP groups occurred between the scales of the left and middle panels of B and C. The right panels show the original cortical surfaces. L denotes the left hemisphere, and R denotes the right hemisphere. **(D)** The asymmetry maps of subjects *i* and *j* at different spatial scales. We projected their left hemisphere onto the right hemisphere to create perfectly symmetric brains. We then calculated the Euclidean distance between the vertices of the original right hemisphere (but also on the fsLR-32k template space) and their projected right hemispheres. Higher Euclidean distances reflect higher degrees of asymmetry. It is clear that subject *j*'s brain is more asymmetric than subject *i* across spatial scales, which is in line with the results in panel A with subject *j*'s MAS values lower than subject *i*'s MAS values.

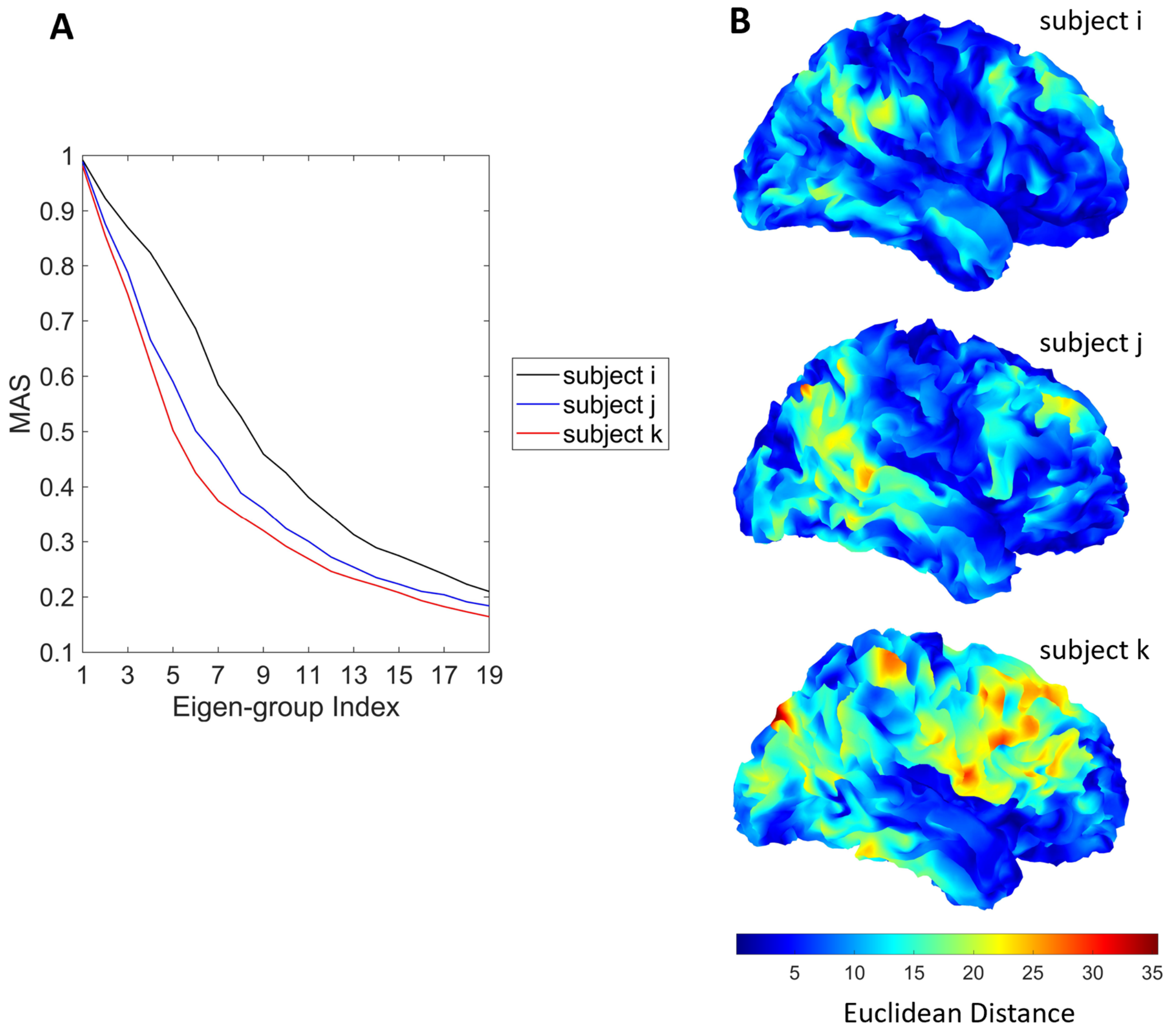

Supplementary Figure 2. Comparison of the excluded patient to other subjects. (A) MAS of the excluded subject (red line; denoted as subject *k*), the most asymmetric brain among the remaining subjects (blue line; subject *j*), and a healthy control subject (black line; subject *i*). The patient with early psychosis (EP; subject *k*) was excluded because this patient's eigen-group-specific MAS across eigen-groups were more than three standard deviations below the sample mean. A lower MAS value represents a higher degree of asymmetry, which means that the brain of subject *k* was much more asymmetric than other subjects. The asymmetry level of subject *k* was much higher than of other subjects, even for the most asymmetric brain (with the lowest MAS; subject *j*) among the remaining subjects. **(B)** Visualisation of the degree of brain asymmetry with Euclidean distance. To demonstrate the degree of asymmetry, we used brain images registered to the fsLR-32k template[2], and we projected their left hemisphere onto their right hemisphere to create perfectly symmetric brains. We then calculated the Euclidean distance between the vertices of the original right hemisphere (also on the template space) and their projected right hemisphere. Higher Euclidean distances reflect higher degrees of

asymmetry. It is clear from panel B that the excluded subject k's brain is more asymmetric than subject j, whose brain is one of the most asymmetric in this study. Note that the main finding of this study is that EP patients' cortex was more asymmetric, i.e., eigen-groups of the MAS were lower, than the healthy control individuals. Thus, if we include this excluded subject, the effect sizes of our study will be even higher, but to prevent inflating our results, we decided to exclude this subject. The exclusion of this subject does not reflect any limitation of our methods.

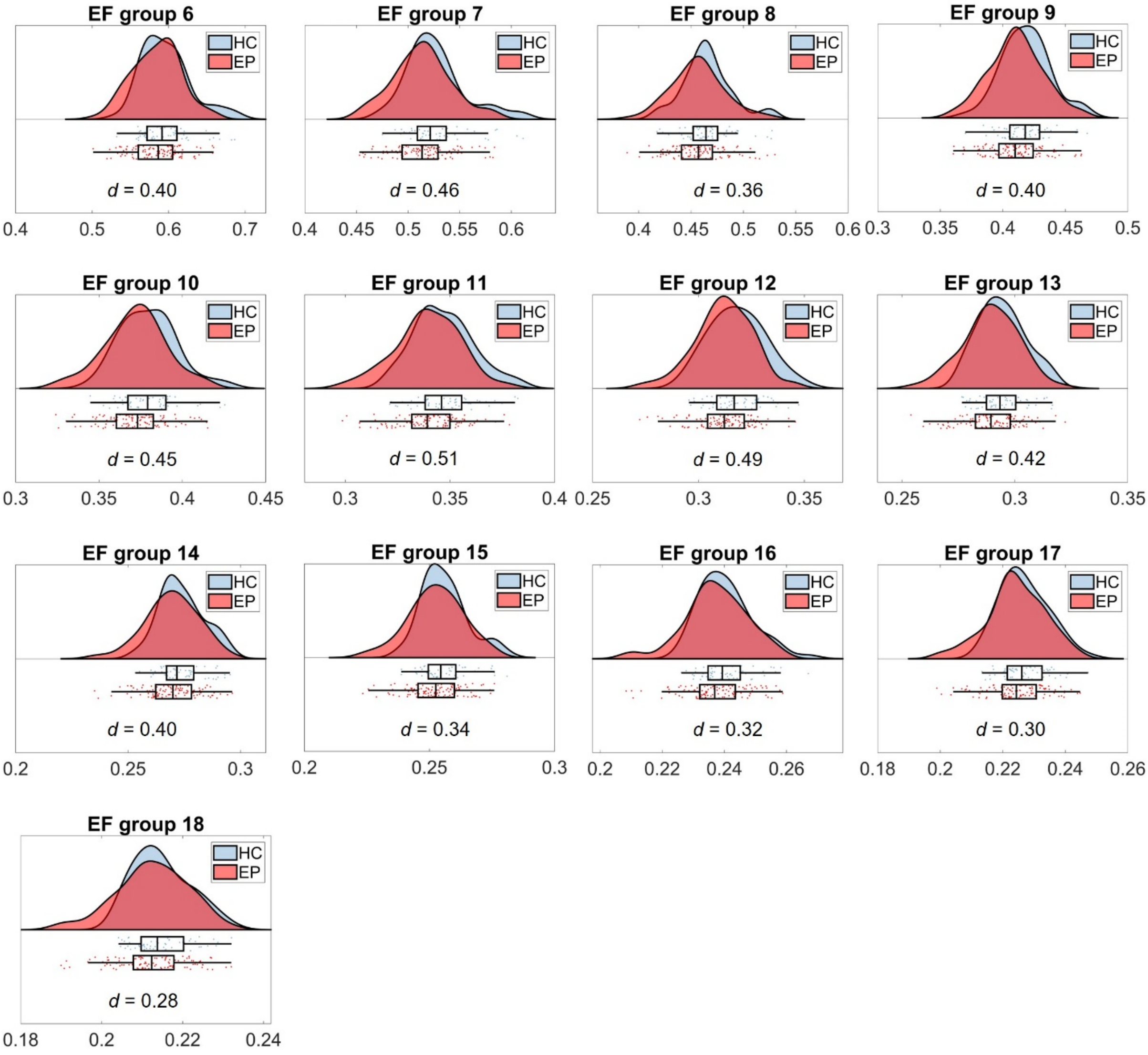

Supplementary Figure 3. Matched asymmetry signatures (MAS) of the 6th to 18th eigen-groups are significantly different between healthy control (HC) and early psychosis (EP) groups. Smoothed distributions and boxplots with mean and interquartile range[3] of the MAS among HC and EP groups. Under these 13 spatial scales, EP patients showed lower levels of MAS, indicating a higher degree of cortical shape asymmetries. The *d* values are effect sizes (Cohen's *d*).

References

1. van der Gaag M, Hoffman T, Remijsen M, et al. The five-factor model of the Positive and Negative Syndrome Scale II: a ten-fold cross-validation of a revised model. *Schizophr Res*. Jul 2006;85(1-3):280-7. doi:10.1016/j.schres.2006.03.021

2. Van Essen DC, Glasser MF, Dierker DL, Harwell J, Coalson T. Parcellations and hemispheric asymmetries of human cerebral cortex analyzed on surface-based atlases. *Cereb Cortex*. Oct 2012;22(10):2241-62. doi:10.1093/cercor/bhr291

3. Allen M, Poggiali D, Whitaker K, Marshall TR, Kievit RA. Raincloud plots: a multi-platform tool for robust data visualization. *Wellcome Open Res*. 2019;4:63. doi:10.12688/wellcomeopenres.15191.1
